# Autonomous Mobile Inspection Robots in Deep Underground Mining—The Current State of the Art and Future Perspectives

**DOI:** 10.3390/s25123598

**Published:** 2025-06-07

**Authors:** Martyna Konieczna-Fuławka, Anton Koval, George Nikolakopoulos, Matteo Fumagalli, Laura Santas Moreu, Victor Vigara-Puche, Jakob Müller, Michael Prenner

**Affiliations:** 1 Faculty of Geoengineering, Mining and Geology, Wrocław University of Science and Technology, 15 Na Grobli Street, 50-421 Wrocław, Poland; martyna.konieczna-fulawka@pwr.edu.pl; 2 Department of Computer Science, Electrical and Space Engineering, Signals and Systems, Luleå University of Technology, 971 87 Luleå, Sweden; anton.koval@ltu.se (A.K.); george.nikolakopoulos@ltu.se (G.N.); 3 Department of Electrical and Photonics Engineering Automation and Control, Technical University of Denmark, Elektrovej, 2800 Kongens Lyngby, Denmark; lsamo@dtu.dk (L.S.M.); s222929@student.dtu.dk (V.V.-P.); 4 Chair of Mining Engineering and Mineral Economics—Conveying Technologies, Montanuniversität Leoben, Franz-Josef-Straße 18, 8700 Leoben, Austria; jakob.mueller@unileoben.ac.at (J.M.); michael.prenner@unileoben.ac.at (M.P.)

**Keywords:** mobile inspection robots, autonomy in underground mining, autonomous robotics, condition monitoring

## Abstract

In this article, the current state of the art in the area of autonomously working and mobile robots used for inspections in deep underground mining and exploration is described, and directions for future development are highlighted. The increasing demand for CRMs (critical raw materials) and deeper excavations pose a higher risk for people and require new solutions in the maintenance and inspection of both underground machines and excavations. Mitigation of risks and a reduction in accidents (fatal, serious and light) may be achieved by the implementation of mobile or partly autonomous solutions such as drones for exploration, robots for exploration or initial excavation, etc. This study examines various types of mobile unmanned robots such as ANYmal on legs, robots on a tracked chassis, or flying drones. The main scope of this review is the evaluation of the effectiveness and technological advancement in the aspect of improving safety and efficiency in deep underground and abandoned mines. Notable possibilities are multi-sensor systems or cooperative behaviors in systems which involve many robots. This study also highlights the challenges and difficulties of working and navigating (in an environment where we cannot use GNSS or GPS systems) in deep underground mines. Mobile inspection robots have a major role in transforming underground operations; nevertheless, there are still aspects that need to be developed. Further improvement might focus on increasing autonomy, improving sensor technology, and the integration of robots with existing mining infrastructure. This might lead to safer and more efficient extraction and the SmartMine of the future.

## 1. Introduction

The global demand for CRMs continues to grow, driven by their essential role in modern technology and clean energy solutions. Non-energy raw materials are indispensable across all industrial sectors and supply chains. CRMs are irreplaceable in clean technologies such as solar panels, wind turbines, electric vehicles, and energy-efficient lighting, making them vital to the EU’s green energy transition and digital economy [1,2]. However, the EU relies heavily on imports to meet its CRM needs. While some domestic production exists, such as that of hafnium and indium, most critical raw materials are supplied by non-EU countries [3]. For instance, China provides 100% of the EU’s supply of heavy rare earth elements, and South Africa accounts for 71% of the EU’s platinum needs. The concentration and low substitution and recycling rates of many CRMs make the EU vulnerable to supply chain disruptions and geopolitical risks [1].

To mitigate these risks and ensure the supply of CRMs, Europe is looking to enhance its recycling and substitution capabilities and to re-excavate domestic mineral resources, including deep underground and abandoned mines. The existing networks of abandoned or suspended mines across Europe have the potential for CRM recovery. However, deep underground mining faces significant technical, safety, and environmental risks. As mines extend deeper, issues such as ventilation, lighting, and dust control become critical, alongside the structural dangers of unstable tunnels and unsupported shafts. Techno-economic setbacks or environmental concerns were the main reasons the mines were abandoned. These mines, ranging from small, shallow shafts to expansive networks covering several square kilometers, are scattered across Europe and beyond [2].

Re-excavating these abandoned mines presents an opportunity not only to recover valuable minerals but also to repurpose these underground spaces for various applications, such as tourism [4], science [5], and potentially resuming mining operations [6]. However, ensuring that these environments are explored safely and cost-effectively is essential before any such initiative can be undertaken. In this regard, advanced autonomous robotic systems offer a promising solution for surveying and data collection in hazardous underground conditions.

One such case is Grecian Magnesite (GM)’s abandoned mine (Athina, Greece) network in central Greece. These old underground mine drives, established between 1832 and 1965, intersect zones of high-quality magnesite mineralization but have been largely left untouched due to the challenges of manual exploration. Today, GM utilizes cutting-edge surveying technologies, such as simultaneous localization and mapping (SLAM) systems, to map these low-profile drives. While this approach has provided precise 3D models and valuable geological data, the process remains labor-intensive, risky, and costly. Autonomous robotic systems could significantly improve efficiency and safety by eliminating the need for human presence in these dangerous environments while reducing operational costs [4].

Under this framework, the potential utilization of an autonomous exploration system in abandoned/suspended underground mines could significantly cut down on exploration costs and render known resources into reserves. At the same time, abandoned/suspended underground mine mapping and exploration could be executed with minimum risks and without requiring the mobilization of experts in harsh and unfavorable environments. Moreover, using existing underground drives for exploration purposes could skyrocket the green transition of mining companies, since intervention works on terrains of high environmental value could be kept to a minimum.

## 2. Autonomous Solutions in Deep Excavation—Path Planning

Autonomous path planning has a significant role in the work and navigation of mobile robots. To allow mobile robots to work fully autonomously, it is crucial to minimize their dependence on humans and enable them to find the best path from their current location to the destination [7]. Even though, in most cases, there are two or more possible routes, the algorithm of path planning should always pick the most optimal one (this paper focuses on localization, not path planning directly). The decision on which path to choose is made depending on the possibility of minimizing time or energy consumption. Many methods of autonomous path planning have been described in the literature. This variety poses difficulties with choosing the right algorithm, especially considering unique work conditions such as the ability to adapt to terrain [8,9,10].

The modern classification of path planning algorithms should focus on their functional features. Before, the division was made based on the dynamic of the environment (online or offline) and its size (local or global). Such a division can generate errors because some algorithms might be suitable for both categories. Nevertheless, some of the earlier classifications [11,12] take this into account and also include aspects like map limitations, the possibility of re-planning, and robot features in their algorithm [13].

**Figure 1:**
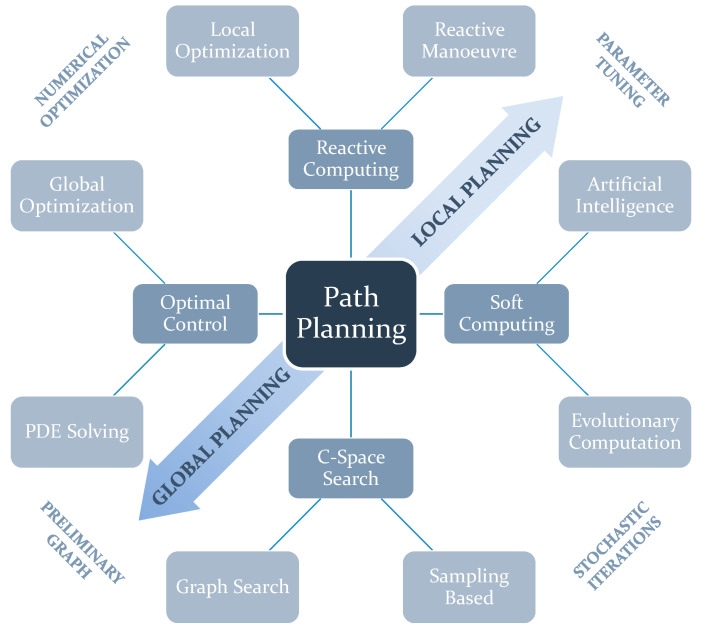
Scheme of autonomous path planning for mobile robots [13].

The classification proposed by Sánchez-Ibáñez [13] is based on four main categories, subcategories, and features that might be common for each of them. The scheme at Figure 1 below mentions the four main categories of autonomous path planning for mobile robots. Each main category is divided into two subcategories. Subcategories next to each other have come features in common, and some of them are more or less close to Global or Local Planning [13].

Exploring recent practical developments specifically tailored to underground mining environments is also essential. Navigation in such a setting is challenged by irregular terrains, dynamic obstacles, low visibility, and the absence of GPS signal.

Graph search and sampling-based algorithms are some of the most used in the research field. A significant number of implementations combine A* with local planners such as DWA (Dynamic Window Approach) [14,15,16], ensuring both an optimal global trajectory and reactive local adjustments, as well as smooth and safe paths. Other researchers modify global planners such as A* or Dijkstra with heuristics (Gaussian filtering [17], articulation angles [18]), reducing computation time and adapting well to non-structured mine tunnels.

More advanced frameworks leverage Reinforcement Learning (RL) methods. For instance, Q-Learning and Deep Q-Networks (DQNs) have been used for learning-based navigation in dynamic underground environments, offering adaptability where traditional model-based planners can fail [19]. A recent approach combines deep RL-based obstacle avoidance with dynamic Voronoi-based multirobot coordination and transfer learning, achieving efficient and safe exploration in dynamic environments [20]. Other hierarchical frameworks integrate global path planning with DRL-based local obstacle avoidance, employing techniques like improved gray wolf optimization (IGWO) and trajectory tracking to achieve both optimality and real-time responsiveness in complex, dynamic scenarios [21].

In conclusion, graph-based methods offer reliability, but they struggle with scalability and dynamic changes. Sampling-based approaches such as RRT* are efficient in complex environments but often require post-smoothing. RL-based methods are more adaptive and robust, though computationally heavier and harder to deploy, requiring edge computing infrastructure.

## 3. Groundbreaking Technologies for Underground Mining Improvement

### 3.1. Identification of Hazards and Inspections of Mining Machines

**Figure 2:**
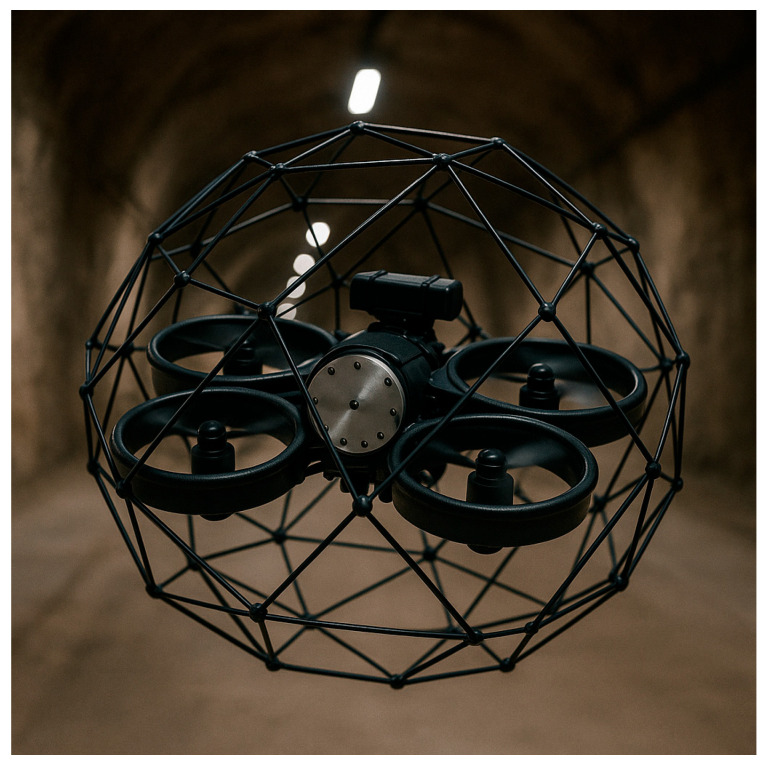
Specially secured drone for mine inspection (based on [33]).

The integration of different types of mobile inspection robots like Micro Aerial Vehicles (MAVs), unmanned ground vehicles (UGVs), or legged robots in the monitoring and diagnostics of underground mining machines has an enormous impact on increasing both safety and efficiency [22,23,24]. These technologies are most often used to monitor many parameters in real time. A significant example is H2020-ROBOMINERS, whose main goal was developing small mining robots capable of moving and navigating in harsh mine environments and making selective mining operations easier [25]. Various solutions, like LiDAR sensors or SLAM (simultaneous localization and mapping) algorithms, cameras, gas sensors, etc., enable robots to operate correctly, detect hazards, improve their autonomy, and be applicable to different kinds of inspections [26]. For instance, MAVs are gaining popularity due to their ability to carry out inspections in low-visibility conditions by using the technique of contour detection [27,28]. Another example is UGVs equipped with thermovision cameras (infrared thermography sensors), extremely useful in detecting overheating components of machines—conveyor belts, for instance—without exposing humans to dangerous factors [29]. Legged robots, such as quadrupedal robots, are designed directly for mining conditions, are able to carry different sensors (temperature, gas, etc.), and are suitable for autonomous work [30,31,32]. When it comes to flying robots, drones can move through difficult areas and collect high-resolution data at the same time like specially secured drone on the Figure 2.

The integration of different types of sensors, such as temperature and gas sensors, allows for detecting gases like methane and carbon monoxide, which provides information about safety in real time and can prevent gas explosions [34,35].

**Figure 3:**
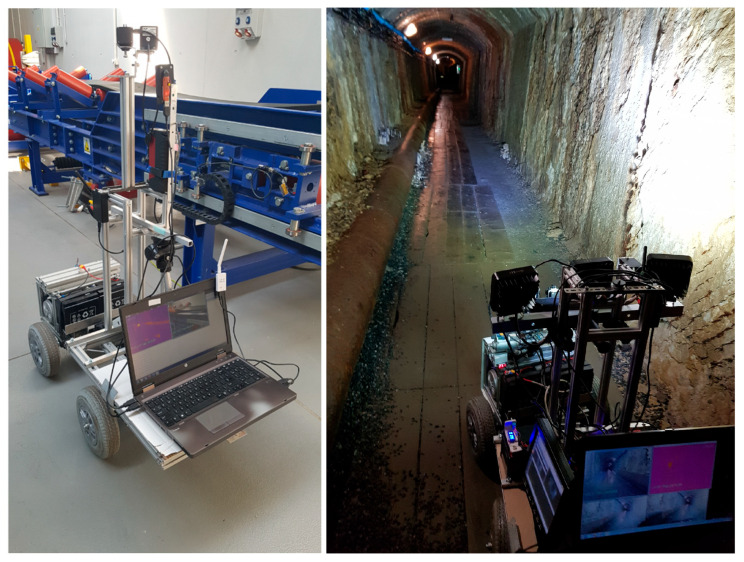
Unmanned ground vehicle at the laboratory on the left [39] and in the field with sensors on the right [29].

A great example of a multi-function mobile robot is the unmanned ground vehicle (wheeled) constructed at the Wroclaw University of Science and Technology (Figure 3). It has the ability to perform 3D mapping of underground tunnels and excavations, and it was also tested for inspections of a Belt Conveyor Idler based on Acoustic Signal-Based Fault Detection [36,37,38]. The platform of the vehicle has a relatively high load-bearing capacity; therefore, additional sensors and measurement systems can be easily added. In the AMICOS Project, a UGV with an installed IR (infrared) camera, RGB (red, green, and blue wavelengths) camera, and stereo camera was used as support in a simulated rescue action conducted in an underground mine [30].

There is a possibility to use various types of mobile robots with different chassis, such as wheeled, wracked (crawler), or walking (legged), and different movement solutions. In this section, examples of walking/moving and flying vehicles are presented, but there are also some swimming solutions used in mobile robots that are important to mention.

**Figure 4:**
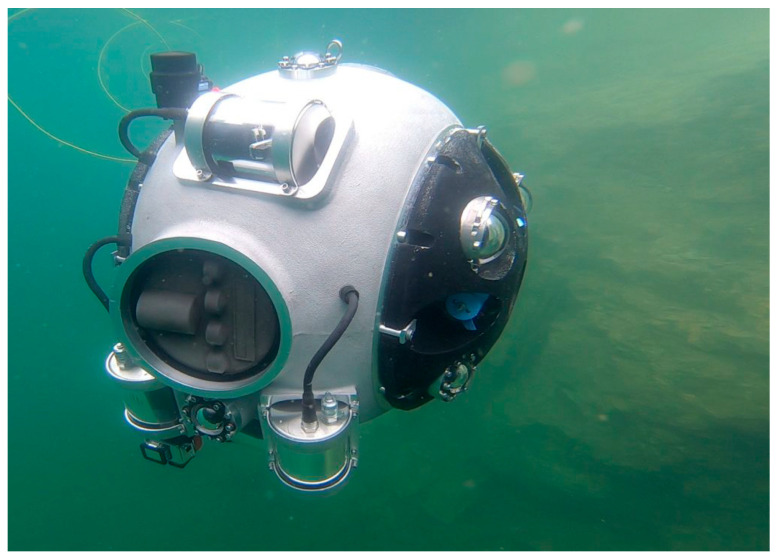
UX-1 underwater vehicle in Kaatiala mine in Finland during in situ research [40].

An example of a swimming robot is the Autonomous Underwater Vehicle, which is able to work fully autonomously in a semi-structured environment (Figure 4). This swimming robot was created to inspect and map flooded underground mine excavations. In these conditions, there is no possibility to communicate with the vehicle, which is why it can perform complex works and navigate fully autonomously even in an unknown environment [40]. Acoustic-based detection devices are successfully used in autonomous robots to detect underwater anomalies such as oil spills. However, they have no application in mining yet, but perhaps they will in the future, especially when combined with other known monitoring techniques used by autonomous robots [41].

All of the mentioned types of robots can be controlled remotely with no need for human presence in dangerous areas, which significantly increases the safety of the crew [41,42]. Maintaining safety standards is one of the priorities nowadays, and conducting real-time monitoring of machine conditions and the ability to detect overheating or even damage to machine elements not only prevents unexpected downtime caused by failure but above all translates into higher safety standards [43].

The described systems are highly adaptable to dynamic conditions in deep underground mining and in abandoned mines as well. Due to machine learning, these robots can operate relatively safely and efficiently in environments which can be unpredictable and hazardous [44]. The use of mobile autonomous inspection robots in underground mining improves efficiency and increases the safety of both machines and humans, reducing the need for human intervention in dangerous areas.

### 3.2. Applications of Mobile Robot Solutions in Deep Underground Mining and Abandoned Mines

To date, autonomous mobile robots have shown significant possibilities in autonomous moving as well as observing, and analyzing the conditions of excavations or machines at the same time. For instance, to explore an abandoned mine or monitor excavations in an underground mine, unmanned mobile robots or autonomous legged robots are used. Nowadays, micro air vehicles (MAVs) are also receiving more attention due to their ability to perform autonomous navigation and inspections in poor-visibility conditions [28,43].

The Groundhog robot [45] was one of the first autonomous ground-based robots designed to explore abandoned mines. Its deployment in the Mathies mine marked a significant achievement in autonomous subterranean exploration, as it could operate in harsh, hazardous environments where wireless communication was impossible. Using a SLAM-based system, Groundhog successfully generated 3D maps of these mines, providing a valuable tool for hazard assessment and safe intervention planning. However, partially flooded mines limited the robot’s operational range.

The UX-1 robot, developed as part of the UNEXMIN project [46], has demonstrated significant value in flooded underground mines. It is a semi-autonomous robot equipped with several sensors, such as multispectral cameras, gamma-ray counters, and sonar systems, with which it can gather geological and mineralogical data from flooded examples. UX-1 explored, for example, the Ecton Mine, which was flooded between 1856 and 1858, and the flooded parts had never been surveyed before.

**Figure 5:**
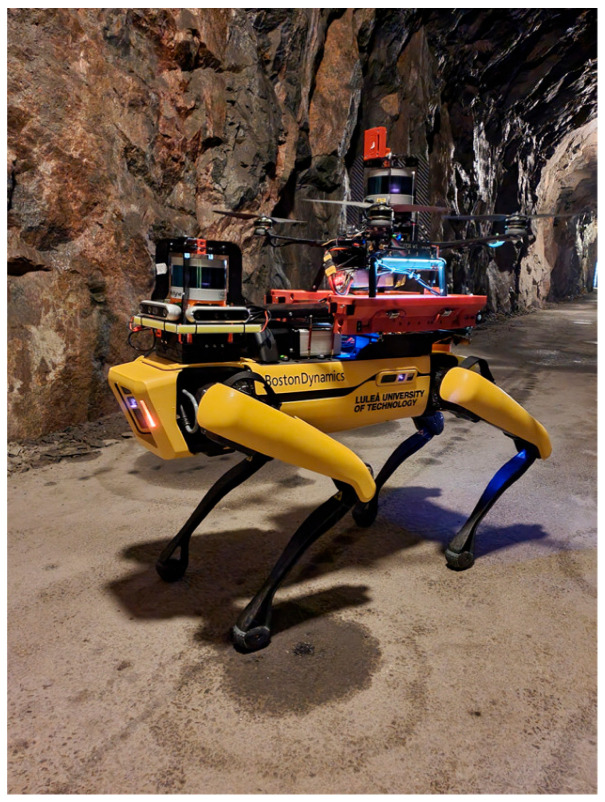
Mixed (legged–aerial) autonomous system [47].

In addition, multimodal robotic systems that integrate ground and aerial capabilities have been developed for subterranean missions (Figure 5) [47]. This solution was developed for underground search-and-rescue reasons. Still, the capability of these systems, like high mobility and agility, long-term operation, and payload, makes these autonomous units very flexible and versatile for other applications, including penetration and mapping of the old workings and other underground structures like shafts, for instance.

**Figure 6:**
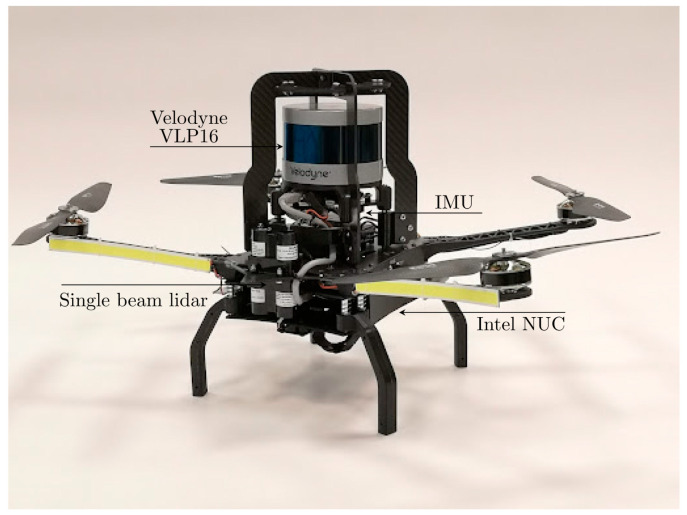
An autonomous drone for mine inspection [50].

In recent decades, huge progress has been observed in the development of drone technology [48]. In many situations, unmanned aerial vehicles (UAVs) seem to be a good solution for examining underground workings [2]. Similar solutions were developed in the NEXGEN SIMS project [49], in which drones were equipped with advanced sensors that allow them to conduct inspections even in areas with poor visibility and difficult accessibility. The drone developed by LTU in the NEXGEN SIMS project is shown in Figure 6.

Mining companies also include robotics in real applications. One use case was developed in collaboration with Rajant, PBE Group, and Australian Droid & Robot [50]. A limestone mine collapse in the Southeastern United States destroyed the mine’s communication infrastructure, making manual inspection impossible. ADR’s Explora XL unmanned robots, equipped with LiDAR and high-definition cameras, were used to navigate debris and unstable environments, transmitting real-time data via Rajant Kinetic Mesh networks. The LiDAR created 3D models of the mine, allowing remote engineers to assess the damage. This approach not only ensured safety by keeping personnel out of hazardous areas but also facilitated a quick inspection, enabling remediation efforts within a week.

It should also be noted that these autonomous and automated platforms can also be equipped with many types of sensors. All these technologies are developing very fast and extending their capabilities in many facets, including long operational time, mobility, agility, autonomic abilities, data collection, and real-time analysis [48]. All these features are often supported by AI usage, etc. It also should be noted that, together with technology development, the costs of those kinds of devices are decreasing; hence, their usage can be commonplace in the near future.

In addition to perception and mobility, underground mine exploration requires physical interaction with the environment. To this end, dedicated manipulator modules are often fitted to a robot. Robotic arms with multiple degrees of freedom (DOFs) and specialized end-effectors (e.g., sampling scoops, grippers, or drilling tools) are used. These manipulators must balance payload capacity (to handle rock or debris), precision (for targeted sampling), and ruggedness (to withstand dust, moisture, and other hazards) while keeping overall system weight low and the power draw within the limited energy budget of mobile platforms. Recent work has demonstrated modular manipulators with quick-swap end-effectors that can, for instance, collect geological samples, deploy environmental sensors, or clear small obstacles, greatly extending the functional scope of underground robotic uses [51,52].

## 4. Multi-Machine Real-Time and Infrastructure-Free Mapping and Localization

**Figure 7:**
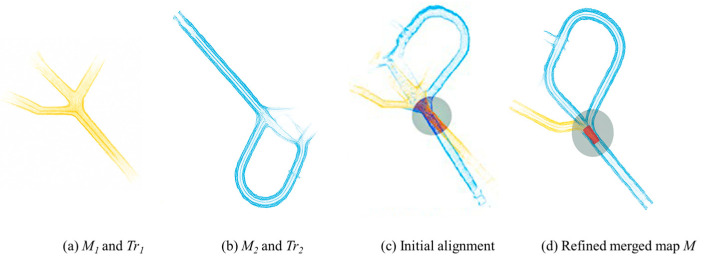
Merging of two maps of the same area [53].

Multi-machine operation in GNSS-denied or GPS-denied subterranean mining environments requires precise localization and mapping [31]. However, taking each machine as an individual unit or agent will not allow us to gather holistic information about the tunnels, especially considering that each machine has limited resources (e.g., battery capacity). As such, there is an obvious need to establish a map-merging framework. Such a framework will allow us to combine multiple local maps from individual machines into one global map. This capability is essential for the next generation of mining machines and for live digital twins of mines. An example of the merging of two maps of the same area is shown in Figure 7.

LiDARs became SoTA sensors for navigation in dark subterranean environments. As output data, these sensors produce a point cloud that, together with IMU fusion, is used in simultaneous localization and mapping (SLAM) algorithms for solving localization and mapping tasks. The methodology that will be developed in PERSEPHONE will focus on the utilization of LiDAR SLAM-produced maps in collaborative multi-agent map merging. This will enable the collaborative building of a comprehensive map of the entire mine. The map will be continuously updated by each machine as it explores the environment. This will ensure the overall mine map reflects the latest information. The machines will share sensor data and map information with each other through a local mesh network (independent of Wi-Fi or similar infrastructure). If a surface connection is available, the updated mine map, information about non-traversable areas, and machine positions will be streamed to surface control centers. This will allow human operators to monitor the operations in real time.

As input data for the newly developed methodology, the following data structures will be required:

(a) Point cloud map of the area.
(b) LiDAR scan.
(c) Radar scan.
(d) Inertial Measurement Unit (IMU) data.
(e) Other parameters.

Considering the core research tasks in path planning, it is assumed that it will focus on collaborative SLAM for safe and efficient navigation of autonomous machines in infrastructure-free environments.

### 4.1. Infrastructure-Free Communication for Multi-Robot Systems

In a telecommunication infrastructure-free environment, such as an abandoned mine where an autonomous exploration mission is being carried out, it is not possible to rely on an existing network infrastructure, and it is not even possible to deploy a temporary wireless network. Therefore, the adoption of an on-board-based fully wireless mesh network is needed, combined also with the adoption of a data buffer where the data is stored during connectivity outage, allowing this information to then be shared with the other elements of the swarm when connectivity is restored.

**Figure 8:**
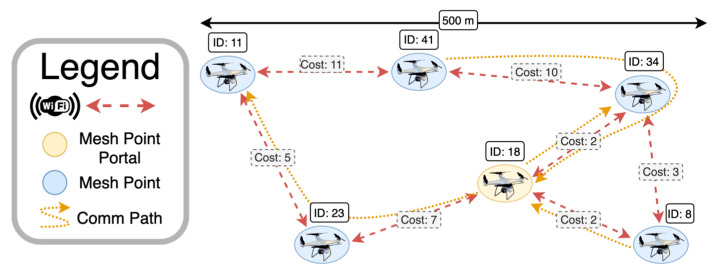
Infrastructure-free communication in a UAV swarm enabled by an on-board Wi-Fi IEEE 802.11s mesh network [54].

In order to achieve these results in a critical environment, it can be possible to exploit the concept of wireless reconfigurable mesh networks, which can be applied in Wi-Fi equipment installed on the nodes of the swarm, as depicted in Figure 8, where a swarm of UAVs is connected through a Wi-Fi IEEE 802.11s mesh network, thus enabling robust infrastructure-free data exchange with the best routing rules based on path cost metrics.

In order to achieve such results in an underground environment, several wireless communication protocols can be combined and implemented on-board of the robotic platforms, with the highest-throughput task delegated to the robust and short-range Wi-Fi IEEE 802.11s mesh, while for less demanding applications, long-range efficient protocols such as LoRa or XBee can be exploited, in either a point-to-point or mesh network configuration.

Furthermore, data buffering capabilities might be added to the platforms, allowing them to store data during critical operations in parts of the tunnels where there might not be any communication availability with other machines, thus allowing them to exchange the gathered data when the connectivity is restored.

Overall, this methodology will be part of the overall Guidance, Navigation, and Control (GNC) framework (KET 3) developed in the project.

This methodology will be applied to the developed concepts of mining machines in order to enable their autonomous and safe navigation. One of the project activities is focused on developing the digital twin of a mine. The utilization of real mining data will allow us to validate the developed methodology in high-fidelity simulations.

A new methodological framework will be validated via the utilization of novel designs of mining machines and real mine data for enabling precision navigation. The aim is to extend existing path planning solutions and make them capable of addressing existing mining challenges.

### 4.2. Multi-Robot Collaboration

Effective multi-robot collaboration is essential to scale inspection, mapping, and exploration tasks in underground mining environments. While prior sections have discussed real-time map merging and infrastructure-free communication, this subsection outlines algorithmic frameworks that enable coordinated task execution and data sharing among autonomous robots.

One approach to coordination is distributed control, where each robot makes local decisions based on shared information or limited communication with peers. Distributed consensus algorithms are a common method, ensuring that robots agree on shared variables such as global pose estimates or frontier targets for exploration [55]. These approaches are inherently robust to node failures and communication dropouts, frequent issues in underground environments.

A more adaptive class of methods is Multi-Agent Reinforcement Learning (MARL). In MARL, each robot (agent) learns a policy through trial-and-error interaction not only with the environment but also with other learning agents. This setup reflects the complexity of real-world applications like underground mining, where multiple autonomous systems must coordinate under conditions of limited observability and dynamic changes. Among the most widely used algorithms are MADDPG (Multi-Agent Deep Deterministic Policy Gradient) [56] and QMIX [57]. Both follow the Centralized Training with Decentralized Execution (CTDE) paradigm [58], where agents are trained with access to global information but act independently at runtime—an approach well-suited to communication-constrained environments such as underground tunnels. As summarized in the survey by Gronauer and Diepold [59], MARL provides strong capabilities in terms of coordination, adaptability, and robust decision-making in cooperative settings.

Another critical aspect of collaboration is task allocation. Strategies such as market-based methods (e.g., auctioning tasks), role-based assignments, or frontier partitioning (e.g., Voronoi-based methods) allow robots to distribute goals efficiently and minimize redundant exploration. Dynamic Voronoi partitioning combined with deep Reinforcement Learning has recently shown strong performance in multi-robot exploration and obstacle avoidance, even under environmental uncertainty [20].

Despite these advances, several challenges remain. Communication constraints can limit synchronization and increase the risk of map misalignment. Inconsistent localization across agents can affect data fusion, and many MARL techniques require significant computational resources or training time, which limits real-time onboard deployment.

## 5. Teleoperation and Digital Twin Technologies

The use of teleoperation and digital twin technologies in underground mining enables enormous progress and development, especially in the aspect of autonomous machines. Teleoperation technologies allow operators to remotely operate machines in difficult mine conditions and even in potentially dangerous areas [60]. In the case of autonomous inspection robots, they allow, for example, for the inspection of conveyors located in workings not intended for human work, with poor or no ventilation. They allow for the inspection of excavations in the event of rescue operations. They increase the safety of both people and machines [61]. Another technology that accelerates the development of intelligent mines and autonomous machines is digital twinning. Creating digital twins means creating virtual copies, or prototypes at the design stage, of machines or entire machinery systems, monitoring their operating parameters and technical conditions in real time, and predicting how changes in external factors or operational parameters will affect these systems [62,63].

**Figure 9:**
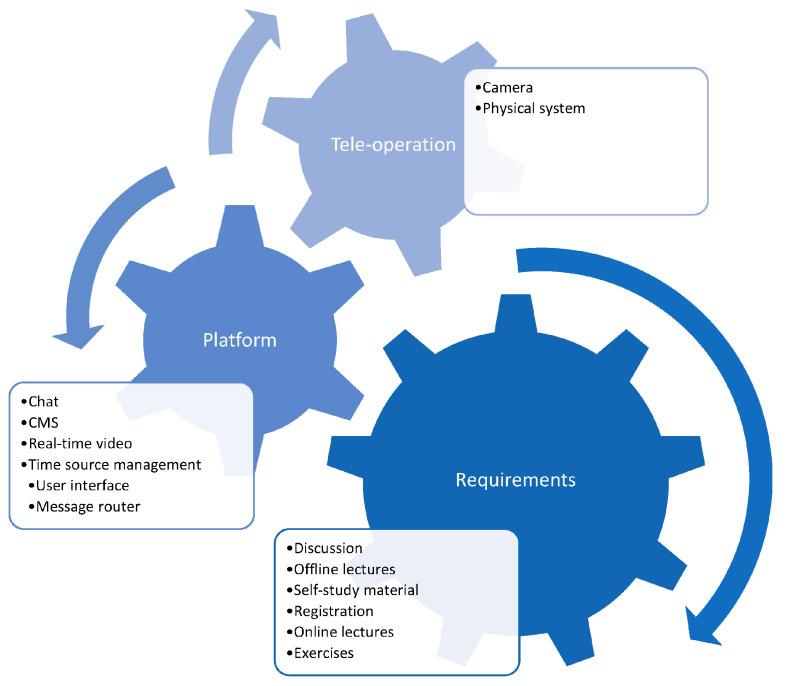
Scheme describing user requirements, services providing required functionalities, and connections between platform services [63].

The scheme shown in Figure 9 describes the user requirements, functionality requirements, and connections between platform services [63].

Teleoperation systems for controlling industrial robots are most often used for tasks that may be difficult or dangerous for humans. In underground mines, the autonomous operation of machines or diagnostic robots also increases employee safety [63]. It is possible to use a lightweight MQTT protocol, thus enabling the remote control of autonomous robots from a considerable distance [64]. It is very important to use video recorders with very low image transmission delay to enable precise work and to view the actual state in real time. Any delays can result in control errors. The Web Real-Time communication project (WebRTC) [65] allows for two-way communication in almost real time and requires the use of a special overload control algorithm. In terms of underground mines, communication problems, poor lighting or no lighting, and difficult mine atmosphere conditions (dust, high humidity) may still pose challenges. All of this may lead to problems with the remote operation of machines and devices.

Digital twinning technology enables the creation of a virtual model, in the analyzed case, of a machine, reflecting its entire life cycle, from design to the actual working machine. The virtual replica of the machine or entire system has an automatic data flow, which must be bidirectional. This allows for monitoring, control, and possible interaction in real time. Digital twins, through the use of teleoperation, enable users to remotely control machine systems. Perfect synchronization and interaction are necessary for proper operation. The third generation of this technology is currently being implemented, integrating AI and deep machine learning. This enables fully autonomous operation of machines that can make decisions in real time. An aspect that may be questionable and is certainly worth developing is cybersecurity. Data exchange between physical and digital twins is based on standards such as OPC UA and, most often, MQTT messaging protocols. Although they were not created with cybersecurity in mind, the latest versions support encryption and authentication algorithms, so they allow for the creation of secure digital twins [63].

A great example of research on and implementation of new technologies is the program implemented by Rio Tinto, “mine of the future”. The mine is testing the possibilities of systems using digital twins to control working systems, increasing efficiency and safety. The company implements technologies such as robots, lasers, and remote-controlled trucks in the Gudai-Darri mine. Based on historical archived data, a replica of the systems operating in the mine was created. Employees are trained on 3D models without having to be on the mine premises. Employees are equipped with tablets and have constant access to all necessary data and documents. The program assumes the permanent introduction of autonomous trucks and machines controlled remotely by employees located in remote offices [66].

## 6. Perspectives on Further Development of Autonomous Solutions for Mining

The role of autonomous mobile robots in mining is significant, and the future is ongoing as well, and it will focus on increasing the capabilities of systems. One of the areas under development is collaborative multi-sensors and many robot systems. Increasing the safety of robots and their sensors or combining the advantages of different types of drive and moving techniques are also developing fields of research [67]. Most often, robots are designed to operate in tandem, which improves the exploration efficiency and the possibilities of data collection accuracy in complex mining environments. Advances in artificial intelligence and machine learning [67,68] are powering the next generation of autonomous robots, which will enable them to quickly adapt to new, harsh conditions and make real-time decisions [20]. These technologies are continuously evolving and have a key role in creating the future of safe and efficient underground mining operations in the Smart Mine of the future.

## 7. Discussion and Conclusions

Currently, the demand for critical raw materials is growing critically, and exploitation is often carried out at greater and greater depths, in increasingly difficult conditions. This is why it is so important to develop existing solutions and look for new ones that enable us to increase the efficiency and safety of machines and, above all, of people. The use of intelligent mobile robots—driving, legged robots and flying drones—in various aspects and for various purposes has become a reality.

Using robots provides measurable benefits in terms of inspecting excavations and machines, collecting data, and even making decisions in conditions dangerous to humans or even in inaccessible places [69,70]. Monitoring the operation of machines or the conditions in the mine in real time significantly improves mining efficiency and work safety [32,71]. Autonomous solutions are necessary to create and operate an intelligent mine; their future development will involve machine learning, creating digital twins, the cooperation of many robots (even of different types), and improvement in the sensors used. A recent significant problem, which was the operation of robots in an environment without access to GPS, has been overcome by solutions like LiDAR sensors or SLAM algorithms [72,73]. All of these developments in the use of autonomous inspection robots may enable the exploitation of deposits that have so far been inaccessible for safety reasons or whose exploitation was not economically profitable [74,75,76]. In a wider perspective, this may mean that the mining of the future will be safer and more efficient, will enable a more rational management of resources, and, consequently, will have a less negative impact on the environment.
